# Diet during pregnancy: Influence of social characteristics and migration in the ELFE cohort

**DOI:** 10.1111/mcn.13140

**Published:** 2021-02-02

**Authors:** Manik Kadawathagedara, Namanjeet Ahluwalia, Marie‐Noelle Dufourg, Anne Forhan, Marie Aline Charles, Sandrine Lioret, Blandine de Lauzon‐Guillain

**Affiliations:** ^1^ Université de Paris, CRESS, INSERM, INRAE Paris France; ^2^ UREN, Faculty of Medicine University of Paris 13 Paris France; ^3^ INED, INSERM, Joint Unit Elfe Paris France

**Keywords:** acculturation, birth cohort, food frequency questionnaire, maternal diet, migrant, pregnancy

## Abstract

Better adherence to dietary guidelines during pregnancy is supposed to result in healthier perinatal outcomes. We aim to characterize the diets of pregnant women by hypothesis‐driven and exploratory approaches and describe potential social determinants. Analyses included 12 048 mothers from the French nationwide ELFE birth cohort. Dietary intake over the last three months of the pregnancy was assessed by a food frequency questionnaire. Two hypothesis‐driven scores (the Diet Quality score, based on benchmarks derived from the National Health and Nutrition Program Guidelines, and the PANDiet score, based on nutrient intake) were calculated. Exploratory dietary patterns were also identified by principal component analysis. Multiple linear regressions were used to assess associations of maternal social characteristics with dietary patterns, accounting for the possible effect modification by their migration status. Five dietary patterns were identified: the Western, Balanced, Bread and toppings, Processed products, and Milk and breakfast cereals. Younger maternal age, single motherhood, unemployment and the presence of older children in the household were related to a suboptimal diet during pregnancy. The less acculturated the women were, the healthier and less processed their diets were, independent of their socio‐economic position. Several social determinants of the quality of women's diets were however moderated by their migration status. These findings shed light on the relations between indicators of social vulnerability, such as single motherhood and unemployment, and poorer diet quality. Given the reduced diet quality that accompanies the acculturation process, it is of paramount importance to identify the specific factors or obstacles that affect migrant women in maintaining their diet quality advantage over the majority population.

**Key messages**

- Beyond the well‐known association of maternal education level and age with diet quality, these findings underline the relations of some indicators of social vulnerability, such as single motherhood and unemployment, with poorer diet quality during pregnancy.
- The less acculturated pregnant women were, the healthier and less processed their diets were, regardless of their socioeconomic position.
- Several demographic and socioeconomic determinants of the quality of women's diets were moderated by their migration status.

## 1 INTRODUCTION

Maternal diet is the major prenatal source of nutrients and can thus influence embryonic, placental, and fetal growth (Blumfield, Hure, Macdonald‐Wicks, Smith, & Collins, 2013). Accordingly, maternal diet during pregnancy appears to be crucial for developing fetuses and their later‐life health status (Abu‐Saad & Fraser, 2010; McMillen et al., 2008). In particular, depletion of the fetal environment during pregnancy can induce a fetal adaptive response of the body, that prioritizes the growth of vital organs such as the brain over the development of others and leads to metabolic alterations (Hales & Barker, 2001). These adaptations are assumed to be especially harmful when followed by overnutrition of the newborn in the postnatal period. Beyond insufficient or excessive energy intake (Blumfield, Hure, Macdonald‐Wicks, Smith, & Collins, 2012), the current problem in high‐income countries is that of unbalanced diet and suboptimal micronutrient intake (Blumfield et al., 2013), especially in disadvantaged populations (Bertin et al., 2016; Lioret et al., 2012; Roberts, Cavill, Hancock, & Rutter, 2013).

Among the latter, cultural and ethnic minority groups, defined as migrants or population subgroups of migrant or indigenous background (Osei‐Kwasi et al., 2016), are reported to have a higher prevalence of diet‐related noncommunicable diseases, along with a suboptimal diet, compared with the native‐born population (Faskunger, Eriksson, Johansson, Sundquist, & Sundquist, 2009; Patel et al., 2006). Beyond socio‐economic position, various sociocultural factors have been shown to have a greater influence on dietary behaviours in cultural minority groups, including acculturation, a multidimensional process in which individuals adopt characteristic ways of life from the culture they are now living in that differ from the primary learning of their cultural background (D'Souza, Jayaweera, & Pickett, 2016; Osei‐Kwasi et al., 2016). In their reviews, D'Souza and colleagues (D'Souza et al., 2016) noted that traditional pregnancy diets vary from society to society, and Urquia and colleagues (Urquia et al., 2010) noted that the association between foreign‐born status and birth outcomes also varies and depends on both migrant subgroup and destination country.

Initiated in 2001, the French National Nutrition and Health Program (PNNS) aims to improve the health status of the population by acting on one of its major determinants: nutrition (S. Hercberg, Chat‐Yung, & Chauliac, 2008). This programme includes guidelines on dietary intake and physical activity for the general population, along with guidelines for specific populations, such as pregnant women. Although several national surveys have monitored dietary intake and adherence to dietary guidelines in the French adult population (Bertin et al., 2016; Castetbon et al., 2009; Dubuisson et al., 2010; Volatier, 2000), these studies included too few pregnant women to assess their adherence to these recommendations. Assessing dietary patterns of pregnant women with specifically designed studies is an important public health objective, to make it possible to identify the groups at greatest risk so that they can receive more tailored information and interventions.

Two main classes of methods are used to derive dietary patterns: first, the production of diet quality scores or indices based on a priori benchmarks from nutritional guidelines or on nutrient intake, and second, the use of exploratory statistical methods, such as principal component analysis and cluster analysis (Kant, 2004). The latter a posteriori data reduction techniques are commonly used in nutritional epidemiology to summarize the variation in food intakes into a small number of patterns and have shown good reproducibility across studies (Newby & Tucker, 2004).

The objective of this study was to characterize the diet of pregnant women living in France, by using both hypothesis‐driven (a priori) and exploratory or data‐driven (a posteriori) approaches, and then to assess its association with demographic and socio‐economic characteristics, while its moderation by migration status is considered.

## 2 MATERIAL AND METHODS

### 2.1 Study population

This analysis is based on data from the ELFE (Etude Longitudinale Française depuis l'Enfance) study, a multidisciplinary nationwide birth cohort including 18 329 children born in 2011 in a random sample of 349 maternity units in mainland France (Charles et al., 2019). Enrolment began in April 2011 and took place during 25 selected days, over four waves of 4–8 days each, each wave covering a different season. Infants met the inclusion criteria if they were singletons or twins born after 33 weeks of gestation to mothers aged 18 years or older who were not planning to move outside of metropolitan France in the next 3 years.

Overall, 51% of eligible parents agreed to their child's participation. Data were collected in standardized interviews conducted by trained interviewers and by self‐administered questionnaires. Efforts were made at inclusion to reach women who were not totally fluent in French: in particular, the information letter and consent form were also edited in the three languages most often spoken in France (after French), i.e. Arabic, Turkish and English. The interviews were also conducted in Soninké, Bambara and Wolof.

### 2.2 Maternal diet during pregnancy

#### 2.2.1 Data collection

At the hospital before discharge, the mothers enrolled in the study completed a self‐administered food frequency questionnaire (FFQ) that described their dietary intake over the last three months of the pregnancy. Consumption of 125 food items was collected, including 12 nonalcoholic and 4 alcoholic beverages, with frequency assessed by a 7‐item scale from ‘never’ to ‘more than once a day’. Usual portion sizes for different food types were estimated with photos indicating five possible sizes, derived from the SU.VI.MAX portion book (S. Hercberg, Deheeger, & Preziosi, 2002) for 75 commonly eaten food items; and for the 50 remaining food items, the midportion in the SU.VI.MAX portion book was automatically assigned. After the transformation of FFQ frequency categories into daily frequencies, daily intake was assessed, for each food item, by combining frequency of intake and portion size. Nutrient intakes were then calculated for all women by multiplying the daily intake of each food item by the nutritional values in the SU.VI.MAX nutrient composition database (S. Hercberg, 2006). The 125 food items were then classified into 48 food groups. Women for whom more than 10 FFQ items were missing were excluded. Conversely, when 10 or fewer items were missing, the missing values were imputed by the sample median. Women were deemed likely misreporters of their food intake, and thus excluded, when their estimated total energy intake was less than 933 kcal/day (3rd percentile) or exceeded 5072 kcal/day (97th percentile). Women were also asked to report their frequency of intake of industrially processed foods on a five‐item scale ranging from ‘never’ to ‘always’: reduced‐sugar and reduced‐fat products, grouped together in the diet food group; prepacked foods; canned foods; and ready‐prepared dishes.

The pregnant women's adherence to the French nutritional guidelines (ANSES, 2016) was assessed with two complementary a priori approaches. The first, the Diet Quality score, was based on consumption of the main food groups, and the second, the PANDiet score, on a range of nutrient intakes. Calculation of the Diet Quality score therefore used 17 quantitative benchmarks (Table S1) adapted from the PNNS‐GS (National Health and Nutrition Program Guideline Score) (Estaquio et al., 2009). The methodology applied the principle of the percentage of each guideline followed by women, consistent with the approach developed in the MoBa cohort (von Ruesten et al., 2014). The overall score, which can range from 0 to 17, is the sum of the scores obtained on each item: the higher the score, the better the adherence to the national guidelines. Details of this score have previously been published (Kadawathagedara et al., 2017). At the same time, the PANDiet score, previously developed and validated among French pregnant women (Bianchi, Mariotti, Verger, & Huneau, 2016), was calculated. It aims to measure the overall quality of a woman's diet by combining the probability of adequate intake for different nutrients, based on recommendations for women in the third trimester of pregnancy. The PANDiet score ranges from 0 to 100, with a higher score again indicating better nutritional quality.

#### 2.2.2 FFQ validation study

A separate study sought to validate the FFQ. It enrolled participants from maternity clinics during their appointment with their obstetrician in major cities across France. Of the 128 women informed about the study by their physician and asked to participate, 105 consented. Among them, 67 (age: 31.4 ± 4.8 years) could give the study dietician information for at least one 24‐h recall over the telephone (out of three initially planned within the last three months of pregnancy); and 62 women completed the FFQ assessing foods consumed during the last trimester. Participants received the study FFQ by mail a month before the planned due date and a telephone reminder to complete the FFQ within a week after their baby's birth. Participants with an incomplete FFQ (*n* = 4) and those for whom the estimated energy intake by FFQ was considered unrealistic (*n* = 2; i.e. 600 Kcal for one subject and 6000 Kcal for the other subject) were excluded from the analysis. The analysis covered the data from the 56 women who provided at least one 24‐h recall and the FFQ were analysed. The methodology of this validation study is described in greater detail in the Supporting Information, as are its results (Table S2).

### 2.3 Demographic and socio‐economic characteristics

As the demographic and socio‐economic characteristics collected at the two‐month telephone interview were more detailed than at the maternity ward interview, we prioritized the data collected at 2 months when they conflicted. Demographic and socio‐economic characteristics studied were as follows: mother's age at delivery (18–25, 25–29, 30–34 or ≥35 years), older children in the household (none, 1 or more), education level (up to lower secondary, upper secondary, intermediate, 3‐year university degree, at least 5‐year university degree), work status [employed, unemployed, not in the labour force (e.g. housewife, student, disabled, retired)], single motherhood (yes/no) and household income per consumption unit (≤€750, €751–1111, € 1112–1500, €1501–1944, €1945–2500, or >€2500/month, that is, ≤$815, $816–1207, $1208–1630, $1631–2113, $2114–2717, or >$2717/month), city size (rural area, <50 000 inhabitants, 50 000–199 999 inhabitants, 200 000–1 999 999 inhabitants, Paris metropolitan area). Her migration status was defined in three categories: ‘majority population’, which included women born with a French nationality to two French parents (in or outside of France); ‘descendants of migrants’ including women born in France with at least one non‐French parent; and ‘migrants’ including women not born in France, and without French citizenship at birth.

### 2.4 Sample selection

The study excluded infants whose parents withdrew consent during the first year (*n* = 57) (Figure S1). In twin births, random selection of one twin (*n* = 287 exclusions) made it possible to count each mother of twins only once in the analysis. The analyses excluded women who did not complete the FFQ (*n* = 2,013), or with more than 10 missing values (*n* = 658), with implausible energy intake (*n* = 910) or with missing data for use of industrially processed products during pregnancy (*n* = 433); this left 13 881 women for dietary pattern identification.

The complete case analysis, which excluded women with at least one missing value for demographic and socioeconomic characteristics, had a sample of 12 048 women.

Compared with the women included in these main analyses, the excluded women were younger (30.3 ± 5.6 years vs. 31.1 ± 4.7 years, *P* < 0.0001) and more likely to be single (9.8% vs. 3.1%, *P* < 0.0001) or to have been born abroad (19.5% vs. 7.1%, *P* < 0.0001), and less likely to have a Master's degree (17.2% vs. 21.1%, *P* < 0.0001) or to have been employed during pregnancy (56.6% vs. 76.4%, *P* < 0.0001).

### 2.5 Statistical analyses

#### 2.5.1 Exploratory dietary patterns

Exploratory dietary patterns were derived by principal component analysis (PCA, FACTOR procedure; SAS software (Version 9.4, SAS Institute, Cary, NC, USA)) of the 46 standardized food groups consumed by more than half the sample. The number of factors to retain was determined with the diagram of eigenvalues (the Scree plot) and based on the interpretability of the factors (Cattell, 1966; Kline, 1994). A standardized score was calculated for each individual and pattern to quantify the individual's adherence to the pattern.

The association between each of the dietary scores, whether derived by hypothesis‐driven or exploratory approaches, were analysed with Pearson's correlations. Recommendations for interpreting these correlation coefficients are: low 0.1–0.3, moderate 0.3–0.5 and high >0.5 (Cohen, 1988).

#### 2.5.2 Associations with demographic and socio‐economic characteristics

Associations of demographic and socio‐economic characteristics with maternal hypothesis‐driven and exploratory dietary patterns (outcomes) were tested by multiple linear regressions, which included all the demographic and socio‐economic characteristics and were adjusted for variables related to the study design (maternity unit size and season of enrolment). Possible interactions (i.e. effect modifications) between migration status and all demographic and socio‐economic characteristics were tested for their relations with the various dietary patterns.

#### 2.5.3 Sensitivity analyses

To take into account the inclusion procedure and biases related to nonconsent, the data were weighted (Juillard, 2015) in sensitivity analyses. Weighting also included calibration on margins from the state register's statistical data and the 2010 French National Perinatal study (Blondel, Lelong, Kermarrec, Goffinet, & National Coordination Group of the National Perinatal Surveys, 2012) on the following variables: age, region, marital status, migration status, level of education and primiparity. This weighting was calculated for the subsample that completed the FFQ.

In addition to the main analyses of the complete cases, analyses with multiple imputations dealt with missing data for demographic and socio‐economic characteristics (*n* = 13 618). Missingness of each variable was less than 11% (10.6% for household income; 9.5% for maternal education level; 6.9% for maternal migration status; 6.9% for maternal employment; 6.4% for single parenthood; 6.4% for parity; and 0% for all other variables of interest). On the assumption that data were missing at random, five independent datasets were generated with the fully conditional specification method {MI procedure, FCS statement, NIMPUTE option; SAS software [Version 9.4, SAS Institute, Cary, NC, USA]} and pooled effect estimates were then calculated {MIANALYSE procedure SAS software}. The imputation models included all variables of interest after they were ranked in ascending order of missing data. Categorical variables were imputed with multinomial models, ordinal or binary variables with logistic regressions, and continuous variables with linear regressions.

Statistical analysis was performed with SAS software [SAS software [‘SAS (computer program)’, 2013] Version 9.4, SAS Institute, Cary, NC, USA]. The significance level was set at 0.05.

### 2.6 Ethical considerations

Participating mothers provided written consent for themselves and for their child. Fathers signed the consent form for the child's participation when present at enrolment or were informed about their right to oppose it. The Advisory Committee for Treatment of Health Research Information (Comité Consultatif sur le Traitement des Informations pour la Recherche en Santé), the National Data Protection Authority (Commission Nationale Informatique et Libertés) and the National Statistics Council approved the ELFE study.

## 3 RESULTS

### 3.1 Identification of exploratory (PCA) dietary patterns in the ELFE study

Table 1 describes the sample's characteristics.

**TABLE 1:** Sample characteristics (*n* = 12 048): The ELFE study, 2011

	% (*n*)^a^
Maternal age at delivery (years), mean (SD)	31.1 (4.7)
Maternal Master's degree: yes	21.1%
Maternal migration status	
Migrant	7.1%
Descendant of migrant	9.9%
Majority population	83.0%
Single motherhood	3.1%
First child	44.1%
Maternal work status during pregnancy	
Employed	76.4%
Unemployed	11.1%
Not in the labour force	12.5%
Household income per consumption unit (€/month) mean (SD)^b^	1686 (930)
Diet quality score^c^, mean (SD)	12.8 (1.2)
PANDiet score^d^, mean (SD)	55.7 (9.1)

^a^ Numbers are in % unless otherwise indicated.

^b^ This corresponds to $1832 (1011).

^c^ The diet quality score could range from 0 to 17.

^d^ The PANDiet score could range from 0 to 100.

Five dietary patterns were identified that together explained 28% of the total variance. The first pattern was characterized by high positive factor loadings for French fries, fast foods, potatoes, mixed dishes, cakes and pastries, sauce (e.g. mayonnaise or ketchup), sweet snacks, red meat, potato chips and similar products, processed meat, candies and breaded fish (Table 2). It is labelled the ‘Western’ pattern. The second pattern was characterized by high positive factor loadings for cooked vegetables, raw vegetables, fish, fruit, whole‐grain bread, legumes, yogurt and cottage cheese, and dried fruit and negative coefficients for sweetened beverages. It is referred to as the ‘Balanced’ pattern. The third pattern, with high coefficients for honey/jam, chocolate, butter and, to a lesser extent, cheese and bread, is named the ‘Bread and toppings’ pattern. The fourth pattern is labelled the ‘Processed products’ pattern in view of its high coefficients for prepacked foods, ready‐prepared dishes, canned foods and diet foods. Finally, the fifth pattern was characterized by high positive coefficients for milk, cocoa powder, and breakfast cereals and negative coefficients for coffee. It is called the ‘Milk and breakfast cereals’ pattern.

**TABLE 2:** Factor loadings for dietary patterns during pregnancy, derived from principal component analysis (*n* = 13 881): The ELFE study, 2011

	Pattern 1	Pattern 2	Pattern 3	Pattern 4	Pattern 5
French fries	0.54	−0.11	−0.30	−0.11	−0.21
Fast foods	0.51	−0.10	−0.07	0.12	−0.03
Potatoes	0.51	0.14	−0.30	−0.11	−0.16
Mixed dishes	0.50	0.06	−0.30	0.03	0.05
Cakes and pastries	0.49	−0.22	0.24	−0.10	−0.01
Sauce	0.44	−0.21	0.02	0.00	−0.14
Biscuits and chocolate or cereal bars	0.44	−0.24	0.35	−0.07	0.03
Red meat	0.40	0.16	−0.25	−0.02	−0.08
Chips and savoury biscuits	0.40	−0.18	0.09	0.04	−0.16
Processed meat	0.39	−0.04	0.06	0.17	−0.18
Candy	0.38	−0.27	0.25	−0.08	−0.07
Breaded fish	0.38	0.05	−0.24	−0.06	0.04
Dairy dessert	0.34	−0.14	0.23	−0.08	0.13
Other starchy foods	0.31	0.21	−0.23	0.00	0.00
Cream	0.29	0.05	0.21	0.02	−0.08
Chocolate spread	0.27	−0.26	0.25	−0.13	0.06
Ice cream	0.25	−0.08	0.05	−0.06	−0.08
Poultry	0.24	0.23	−0.19	0.06	−0.02
Shellfish	0.19	0.18	−0.13	0.00	0.04
Fruit juice	0.19	−0.08	0.15	−0.11	0.06
Cooked vegetables	0.21	0.60	−0.18	−0.14	0.06
Raw vegetables	0.10	0.56	0.05	0.01	−0.06
Fish	0.19	0.47	−0.11	0.00	0.06
Fruit	0.14	0.47	0.11	−0.17	0.10
Wholegrain bread	−0.07	0.44	0.10	0.06	0.10
Legumes	0.14	0.41	−0.20	−0.15	0.09	
Yogurt and cottage cheese	0.04	0.37	0.23	0.11	0.17
Dried fruit	0.01	0.37	0.13	−0.16	0.11
Tea	−0.06	0.32	0.18	0.11	−0.13
Eggs	0.28	0.32	−0.13	−0.02	0.02
Oil, margarine	0.16	0.30	0.24	0.11	−0.16
Water	0.00	0.26	0.11	0.01	0.13
Sweetened beverages	0.34	−0.36	0.00	−0.02	−0.15
Honey/jam	0.01	0.28	0.49	−0.07	0.02
Chocolate	0.15	0.08	0.48	−0.04	0.11
Butter	0.12	0.19	0.44	0.05	−0.08
Cheese	0.20	0.31	0.32	0.10	−0.04
White bread	0.13	0.11	0.30	0.05	−0.05
Prepacked foods	0.17	−0.08	0.05	0.62	0.34
Ready‐prepared dishes	0.19	−0.11	−0.08	0.59	0.34
Canned foods	0.20	−0.04	−0.05	0.52	0.32
Diet foods	0.06	0.11	−0.11	0.32	0.08
Milk	0.15	−0.08	−0.07	−0.33	0.56
Cocoa powder	0.19	−0.20	0.09	−0.33	0.53
Breakfast cereals	0.14	−0.09	−0.04	−0.16	0.38
Coffee	0.07	0.08	0.09	0.23	−0.40
% explained variance	7.9	6.8	4.7	3.7	3.5
Proposed label	Western	Balanced	Bread and toppings	Processed products	Milk and breakfast cereals

The correlation between the Diet Quality and the PANDiet scores was moderate (0.43, *P* < 0.0001). The correlations of both the Diet Quality and the PANDiet scores with the exploratory Balanced pattern score were respectively high (0.65, *P* < 0.0001) and moderate (0.42, *P* < 0.0001). The correlations of the remaining four exploratory dietary patterns with each of the Diet quality and the PANDiet scores were low, i.e. respectively −0.03 and 0.20 for the Western pattern (*P* > 0.05 and *P* < 0.0001); 0.01 and −0.05 for the Bread and toppings pattern (*P* > 0.05 and *P* < 0.0001); −0.03 and −0.14 for the Processed products pattern (both *P* < 0.0001); and 0.21 and 0.24 for the Milk and breakfast cereals pattern (both *P* < 0.0001).

### 3.2 Demographic or socio‐economic characteristics and maternal diet

Table S3 presents the bivariate associations of the maternal dietary patterns with demographic or socio‐economic characteristics.

***Moderating effects of maternal migration status***

There was no interaction between migration status and any of the variables of interest for the Western, Bread and toppings and Milk and breakfast cereals dietary patterns. Conversely, a moderating effect of the variable ‘migration status’ was observed on the whole in the three healthier dietary patterns (i.e. Diet quality score, PANDiet score and Balanced) for the following factors: maternal education (*P* value for interactions ranging from <0.0001 to 0.046), maternal occupation (*P* value for interactions ranging from 0.0029 to 0.066), and household income (*P* value for interactions ranging from <0.0001 to 0.033). Interaction effects were also observed between migration status and both household income (*P* value = 0.09) and older children in the household (*P* value = 0.03) in their association with the Processed dietary pattern. Consequently, analyses involving the Western, Bread and toppings, and Milk and breakfast cereals dietary patterns used the entire sample, whereas the associations between demographic or socioeconomic characteristics and either of the three healthier or the Processed dietary patterns were assessed separately according to maternal migration status.

***Western dietary pattern***

Maternal age, maternal education level and household income were inversely related to this dietary pattern, while older children in the household, unemployment during pregnancy and single motherhood were positively related to it (Table 3). Maternal migration status was not associated with this dietary pattern.

**TABLE 3:** Multivariate associations between demographic and socio‐economic characteristics and maternal diet for the whole sample (*n* = 12 048): The ELFE study, 2011

	PCA pattern 1: Western		PCA pattern 3: Bread and toppings		PCA pattern 5: Milk and breakfast	
Maternal age at deliver		^***^		^***^		^***^
<25 years	0.22 [0.15; 0.29]^a^		−0.08 [−0.15; −0.01]		0.08 [0.01; 0.16]	
25–29 years	0 [ref]		0 [ref]		0 [ref]	
30–34 years	−0.06 [−0.10; −0.02]		0.13 [0.09; 0.17]		−0.12 [−0.17; −0.08]	
35 years or more	−0.18 [−0.23; −0.13]		0.15 [0.10; 0.20]		−0.22 [−0.28; −0.17]	
Maternal education level		^***^		^***^		^***^
Up to lower secondary	0.31 [0.20; 0.41]		−0.37 [−0.48; −0.26]		−0.20 [−0.31; −0.08]	
Upper secondary	0.22 [0.17; 0.28]		−0.25 [−0.30; −0.19]		−0.17 [−0.23; −0.12]	
Intermediate	0.09 [0.04; 0.14]		−0.14 [−0.19; −0.09]		−0.06 [−0.11; 0.00]	
3‐year university degree	0.06 [0.01; 0.11]		−0.02 [−0.08; 0.03]		0.02 [−0.03; 0.08]	
At least 5‐year university degree	0 [ref]		0 [ref]		0 [ref]	
Maternal migration status				^***^		^***^
Migrant	−0.01 [−0.07; 0.06]		−0.58 [−0.65; −0.51]		0.24 [0.17; 0.31]	
Descendant of migrant	0.03 [−0.03; 0.09]		−0.25 [−0.31; −0.19]		0.03 [−0.03; 0.09]	
Majority population	0 [ref]		0 [ref]		0 [ref]	
Single motherhood		*				
No	0 [ref]		0 [ref]		0 [ref]	
Yes	0.11 [0.01; 0.21]		−0.07 [−0.17; 0.03]		−0.01 [−0.11; 0.10]	
Older children in household		^***^		^***^		^***^
ELFE child is the first child	0 [ref]		0 [ref]		0 [ref]	
At least one older child	0.18 [0.14; 0.22]		0.09 [0.05; 0.13]		−0.17 [−0.21; −0.13]	
Maternal work status during pregnancy		^***^				
Employed	0 [ref]		0 [ref]		0 [ref]	
Unemployed	0.09 [0.03; 0.14]		0.03 [−0.02; 0.09]		0.05 [−0.01; 0.11]	
Not in the labour force	0.05 [−0.01; 0.11]		−0.03 [−0.09; 0.02]		−0.02 [−0.08; 0.04]	
Household income		^***^		^***^		
≤€750/month^b^	0.18 [0.11; 0.26]		−0.26 [−0.34; −0.19]		0.01 [−0.07; 0.09]	
€751–1111/month^c^	0.08 [0.02; 0.14]		−0.14 [−0.19; −0.08]		−0.03 [−0.10; 0.03]	
€1112–1500/month^d^	0 [ref]		0 [ref]		0 [ref]	
€1501–1944/month^e^	−0.05 [−0.09; 0.00]		0.05 [0.01; 0.10]		0.01 [−0.04; 0.06]	
€1945–2500/month^f^	−0.09 [−0.14; −0.03]		0.11 [0.06; 0.17]		0.06 [0.00; 0.12]	
>€2500/month^g^	−0.12 [−0.19; −0.05]		0.10 [0.03; 0.17]		−0.02 [−0.09; 0.06]	

^a^ Values are estimates [95% CI] from multivariable linear regressions, also adjusted for region of residence, maternity unit size and enrolment wave; the global significance was indicated with the following labels: *** for *p* < 0.001, ** for *p* < 0.01 and * for *p* < 0.05.

^b^ Corresponding to ≤$815/month.

^c^ Corresponding to $816–1207/month.

^d^ Corresponding to $1208–1630/month.

^e^ Corresponding to $1631–2113/month.

^f^ Corresponding to $2114–2717/month.

^g^ Corresponding to >$2717/month.

***Bread and toppings dietary pattern***

Maternal age, maternal education level, older children in the household and household income were positively associated with the Bread and toppings pattern (Table 3). Maternal occupation and single motherhood were not associated with it. Migrant women and, to a lesser extent, descendants of migrants, had lower scores on the Bread and topping pattern than the majority population did.

***Milk and breakfast cereals dietary pattern***

Maternal age and older children in the household were negatively related to this dietary pattern (Table 3). Pregnant women of higher maternal education level scored higher on it, and migrant women higher than the majority population. No association was observed with household income, maternal occupation or single motherhood.

***Diet quality score, PANDiet score, and balanced dietary pattern***

**FIGURE 1:**
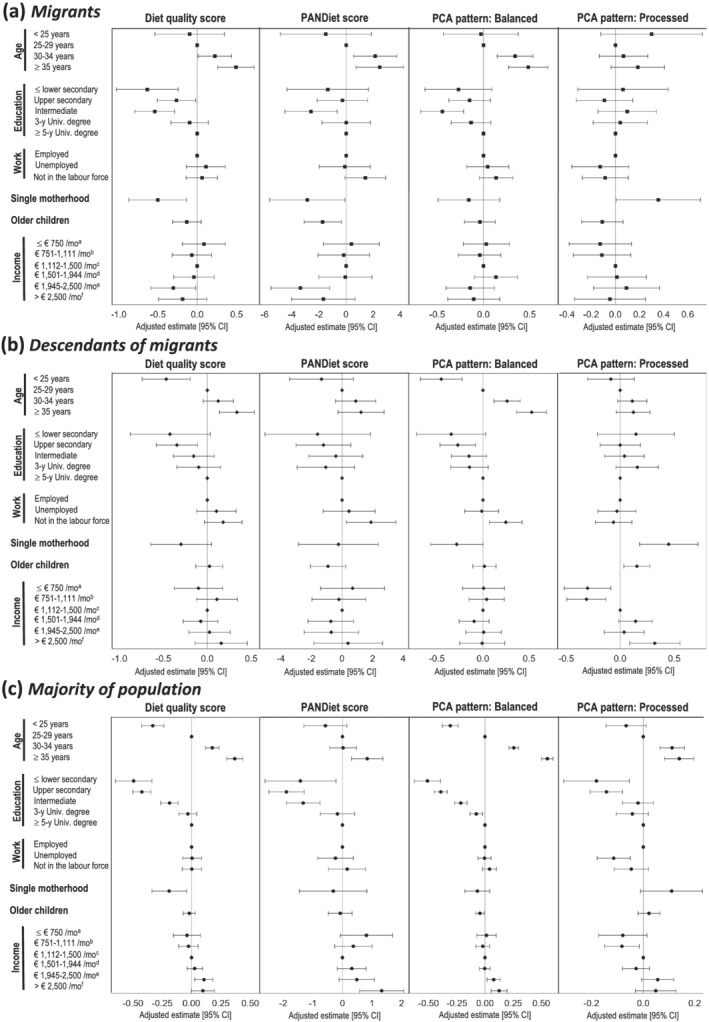
Multivariate associations between demographic or socio‐economic characteristics and maternal diet by maternal migration status: The ELFE study, 2011. Figure 1a presents the multivariate associations among migrants (*n* = 853), Figure 1b those among descendants of migrants (*n* = 1195), and Figure 1c those among the majority population (*n* = 10 000). All multivariable linear regressions presented in this figure were adjusted for region of residence, maternity unit size and recruitment wave. ^a^Corresponding to ≤$815/month; ^b^corresponding to $816–1207/month; ^c^corresponding to $1208–1630/month; ^d^corresponding to $1631–2113/month; ^e^corresponding to $2114–2717/month; ^f^corresponding to >$2717/month

Although positive associations between maternal education and healthy dietary patterns were observed among all women, effect sizes were stronger in the majority population (Figure 1). Household income was positively associated with these patterns only in the majority population. Higher adherence to the PANDiet and Balanced patterns were observed in women not in the labour force only in descendants of migrants; no association was observed between maternal occupation and healthy dietary patterns in either migrants or the majority population. Age was positively related to healthy dietary patterns in all women, regardless of their migration status, and single motherhood, negatively; there was no clear relation to the presence of older children in the household.

***Processed dietary pattern***

None of maternal age, maternal education level and maternal occupation was associated with this dietary pattern (Figure 1). Household income was positively associated with it in the majority population and in descendants of migrants, but not in migrants. The presence of older children was associated with greater adherence to this pattern in descendants of migrants only. Single motherhood was associated with a higher score on this pattern, regardless of migration status.

The analyses based on weighted data or on multiple imputations of missing data produced very similar findings (data not shown).

## 4 DISCUSSION

This study is the first to provide insights into the demographic and socio‐economic determinants of pregnant women's diet in France, while taking the possible moderating effects of migration status into account. Five distinct dietary patterns were identified and labelled as Western, Balanced, Bread and toppings, Processed products, and Milk and breakfast cereals. Overall, older age and foreign cultural background were related to better quality diets during pregnancy, whether quality was assessed by hypothesis‐driven or exploratory approaches. The reverse was true for single motherhood, unemployment, and older children in the household, all of which were associated with suboptimal diets. Maternal education and household income were negatively associated with the Western dietary pattern in the overall population, while their association with the healthier dietary patterns was moderated by the mother's migration status: their traditional positive association with a healthy diet was essentially confirmed in the majority population, and to a lesser extent in mothers from different cultural backgrounds. This acculturation process was also suggested for the Processed dietary pattern, which had higher scores in households with higher incomes, in the majority population and to a lesser extent in descendants of migrants, but not in migrant mothers.

Comparison of the maternal and household characteristics associated with maternal diet in a US study (Bodnar & Siega‐Riz, 2002) showed that a high score on the *Diet Quality Index for Pregnancy* was positively associated with women's age, education, and income. Similar relations between women's characteristics and diet quality during pregnancy have been reported in Norway (Hillesund, Bere, Haugen, & Overby, 2014) and Spain (Rodriguez‐Bernal et al., 2010). The Norwegian study demonstrated that adherence to Nordic or Norwegian dietary guidelines increased slightly with age, education and exercise frequency. Likewise, in the ELFE study, age at delivery and education were positively associated with healthy patterns (i.e. the Diet Quality and the PANDiet scores and the balanced dietary pattern), and negatively with the Western dietary pattern. Interestingly, even after adjustment for education and employment, family income remained negatively associated with the Western dietary pattern and positively associated with the Bread and toppings pattern. These findings are notably similar to those observed among women from the general French population for the relations of age and education level to balanced and healthier dietary patterns (Bertin et al., 2016).

Our findings however further showed that the relation of maternal education to the healthier patterns was strongest in the majority population, lower in descendants of migrants and still lower among migrants. This moderating effect of migration status was also evident for another socio‐economic indicator – household income. On the one hand, it was positively associated with healthy patterns only in the majority population; on the other hand, it was also positively related to the Processed pattern in this population and, to a less extent, in descendants of migrants, but again, not among migrants. Overall, the less acculturated pregnant women were, the healthier and less processed their diet, regardless of their socio‐economic position. The so‐called ‘healthy migrant effect’, which results from a selective process where migrants are on average healthier than, on the one hand, those who stayed behind in the country of origin, and, on the other hand, the majority population born in the destination country (Kennedy, Kidd, McDonald, & Biddle, 2014), might in part explain these findings. It has also been reported that among migrant pregnant women and mothers, cultural factors play a more important role than traditional socio‐economic indicators (such as education and income) (Kumar, Holmboe‐Ottesen, Lien, & Wandel, 2004; Osei‐Kwasi et al., 2016) and result in stronger adherence to their own, apparently healthier, social and cultural dietary traditions. Dietary behaviours are influenced by a variety of factors, such as cultural identity and desire to maintain traditional food identity (which presumably does not include processed foods), religious beliefs and prescriptions, social networks, social norms, and the social role of foods, beliefs and perceptions of healthy food (D'Souza et al., 2016; Osei‐Kwasi et al., 2016). Similarly, previous findings about breastfeeding have underlined that migrant women are more likely to begin by continuing habits from their country of origin and then progressively become acculturated (Kersuzan, Tichit, & Thierry, 2018). It should be noted that the Bread and topping dietary pattern seems to reflect both the traditional bread and jam/honey French breakfast and the bread/cheese third course of traditional French meals, which might explain in part migrant women's lesser adherence to it. Importantly, acculturation, approximated in the current study by the migration status variable, was associated with poorer diet quality (as measured by any of the Diet quality score, PANDiet score, and Balanced and Processed dietary patterns), along with socio‐economic inequalities that emerged in the descendants of migrants. It has been suggested that migration is often accompanied by disruption of social ties and norms, a reduction in socio‐economic position, material hardships, literacy challenges, increased stress and the experience of discrimination – all potential obstacles to maintaining traditional heathy dietary patterns in groups of foreign origin across generations (Allen et al., 2014).

Consistent with other studies (Bodnar & Siega‐Riz, 2002; Hillesund et al., 2014; Rodriguez‐Bernal et al., 2010), single motherhood was related, independently of migration status and socioeconomic position, to lower adherence to guidelines, along with higher scores on the Western and Processed patterns. Similar associations were also observed with unemployment and older children in the household. In a context of higher social vulnerability, obstacles to the availability and accessibility of healthy food, along with time constraints, may partly explain these associations (Dlugonski & Motl, 2013; Mills et al., 2017; Pollard, Kirk, & Cade, 2002) and should inform nutritional interventions targeted at more at‐risk families.

The *Diet Quality* score was constructed to consider adherence to guidelines for all of the core food groups. These groups, however, contributed in different ways to the various dietary patterns derived from PCA: the Western pattern was mainly characterized by meat and fish, and the Balanced pattern by fruit and vegetables. High factor loadings were observed for dairy products in the Balanced pattern (yogurt), the Bread and toppings pattern (cheese), and the Milk and breakfast cereals pattern (milk). These exploratory dietary patterns therefore encompass different dimensions of diet quality.

Only few studies have combined hypothesis‐driven and exploratory approaches to characterize maternal diet. Principal component analysis is a standard technique used in population studies to explore dietary patterns (Bertin et al., 2016; Lioret et al., 2012; Northstone, 2012; Northstone, Emmett, & Rogers, 2008), which takes the potential effect of interactions within combinations of foods and nutrients into account (Newby & Tucker, 2004). While the hypothesis‐driven approach promotes international comparisons and enables the exploration of global adherence to nutritional guidelines, the exploratory approach, which identifies specific patterns within a country and various dimensions of diet quality, may enable the provision of more tailored food‐based recommendations. It should be noted that the two complementary hypothesis‐driven quality scores used in the current study (one derived from food group consumption and the other one based on nutrient intake) were moderately correlated (*r* = 0.43). Both scores were also strongly correlated with the Balanced dietary pattern. These correlations support the external validity of the hypothesis‐driven and exploratory scores.

The ELFE‐FFQ provided reasonably good estimates of food and nutrient intake compared with repeated 24‐h recalls (Table S2); correlation coefficients are similar to those reported in other validation studies among pregnant women (Cheng et al., 2008; Erkkola et al., 2001; Mikkelsen, Osler, & Olsen, 2006; Mouratidou, Ford, & Fraser, 2006; Pinto et al., 2010; Robinson, Godfrey, Osmond, Cox, & Barker, 1996), although a US study noted higher correlations (Brown et al., 1996). There is no general consensus on a ‘satisfactory’ level of correlation. In most validation studies, correlation coefficients between methods are considered poor when <0.30; fair when between 0.3 to 0.49; and good when >0.50 (Hankin, Wilkens, Kolonel, & Yoshizawa, 1991). Adjustment for energy and correction for random intraindividual variation tended to increase the magnitude of the correlation coefficients (Brantsaeter, Haugen, Alexander, & Meltzer, 2008; Brown et al., 1996; Erkkola et al., 2001; Mouratidou et al., 2006). Correlation coefficients are commonly employed and reported in validation studies, but the level of agreement assessed by cross‐classification into quartiles or quintiles of their intake is considered more appropriate to evaluate the ability of an FFQ to rank individuals into low or high intakes relative to the reference method (Garrow, 1995). In the ELFE study, only a very small percentage of the women was grossly misclassified (classified into opposing quintiles in FFQ vs. 24‐h recalls). It should be noted that a review of 90 studies of energy intake during pregnancy in high‐income countries found an average intake of 2340 Kcal in the third trimester, very close to the FFQ estimates reported here (Blumfield et al., 2012). The mean PANDiet score was also very close to the value previously estimated in a population of pregnant women or in any case of childbearing age (Bianchi et al., 2016). It is however recognized that the FFQ was completed after delivery and collected dietary intake retrospectively over the last three months of pregnancy, which does not necessarily reflect the diet across all the pregnancy period.

The ELFE cohort is a nationwide study of births in 2011 in metropolitan France (excluding very premature babies). The main analyses were conducted on the complete case sample. It must also be acknowledged that, as it is often the case in cohorts, women not participating in the cohort or those excluded from the analyses were more strongly socially disadvantaged and more likely to have been born abroad, which is a potential limitation in terms of generalizability of the findings. We also acknowledge that whereas the main documents were edited in the three languages most often spoken in France (after French), this was not done for the FFQ. Even if women could be helped to complete the questionnaire, this limitation probably led to a higher probability of missing dietary information among women with lower literacy in French. Nonetheless, addressing missing data for demographic and socioeconomic characteristics by the multiple imputation method, or running the analysis using weighting to account for selection bias, produced results consistent with those of the main analyses, thus suggesting that selection bias has limited impact on our findings. Furthermore, dietary patterns derived from PCA in the current study were overall consistent with literature based on cross‐sectional studies, for which selection bias is supposedly smaller (Bertin et al., 2016). These findings support the robustness of the results. Two specific limitations about the analysis of the migrant effect must be acknowledged. The first is the selection of migrant women by their ability to read French (which was necessary for them to be able to complete the FFQ); this may have resulted in an overrepresentation of educated women in the migrant group. However, enough variability remained to observe an association with education level for some of the dietary indices. Second, the FFQ may have underrepresented specific foods consumed by migrants. The principal strengths of the ELFE study also include the large sample and the wide range of socio‐economic, cultural and demographic variables collected, which made it possible to disentangle the relation between the various components of social background and diet at the same time that the moderating effect of maternal migration status is taken into account.

## 5 CONCLUSION

The present study used both hypothesis‐driven and exploratory approaches to characterize the diet of pregnant women more comprehensively. In addition to two scores indicating higher adherence to dietary (Diet quality score) and nutritional (PANDiet score) guidelines, five distinct data‐driven dietary patterns were identified and labelled as Western, Balanced, Bread and toppings, Processed products, and Milk and breakfast cereals. These findings from the large, nationwide ELFE birth cohort suggest that younger maternal age, single motherhood, and unemployment – all indicators of greater social vulnerability – as well as the presence of older children in the household are determinants of a suboptimal diet during pregnancy. The consideration of migration status makes it possible to show that the less acculturated pregnant women were, the healthier and less processed their diets were, independent of socio‐economic position. Several demographic and socio‐economic determinants of the quality of women's diets were moderated by their migration status. These original findings should inform the development of future nutritional interventions during pregnancy. Such interventions would be more efficient and likely more effective if directed at more socially disadvantaged populations. Given the reduction in diet quality that appears to accompany the acculturation process, it is of paramount importance to identify the specific factors or obstacles that affect migrant women in maintaining their diet quality advantage over the majority population and thereby prevent the emergence of diet‐related health inequalities for themselves and their offspring.

## CONFLICTS OF INTEREST

The other authors had no conflicts of interest relevant to this article to disclose.

## CONTRIBUTIONS

MK conceptualized and designed the study, conducted statistical analyses, drafted the initial manuscript, and approved the final manuscript as submitted; MND and AF designed the data collection instruments, supervised data collection, critically reviewed the manuscript, and approved the final manuscript as submitted; NA, MAC, SL and BLG conceptualized and designed the study, contributed to data analysis and interpretation of the study, reviewed and revised the manuscript, and approved the final manuscript as submitted.

## Supporting information


**Figure S1.** Participant flow chart
**Table S1.** online. Benchmarks used for the Diet Quality score
**Table S2.** Consistency between nutrient or food group intake estimated by both the ELFE food frequency questionnaire and by 24‐hour recalls (n = 56)
**Table S3.** Bivariate associations between familial characteristics and maternal diet during pregnancy (*n* = 12,048): the ELFE study, 2011

## Data Availability

The data underlying the findings cannot be made freely available for ethical and legal restrictions imposed, because this study includes a substantial number of variables that, together, could be used to re‐identify the participants based on a few key characteristics and then be used to have access to other personal data. Therefore, the French ethics authority strictly forbids making these data freely available. However, they can be obtained upon request from the ELFE principal investigator. Readers may contact marie-aline.charles@inserm.fr to request the data.
