# Supplementary material for: Diet during pregnancy: Influence of social characteristics and migration in the ELFE cohort
Source: Matern Child Nutr. 2021 Feb 2;17(3):e13140. doi: 10.1111/mcn.13140 (PMC8189248; doi:10.1111/mcn.13140)
Supplement: Supplementary file 1 — Figure S1. Participant flow chart Table S1. online. Benchmarks used for the Diet Quality score Table S2. Consistency between nutrient or food group intake estimated by both the ELFE food frequency questionnaire and by 24‐hour recalls (n = 56) Table S3. Bivariate associations between familial characteristics and maternal diet during pregnancy (n = 12,048): the ELFE study, 2011 [file MCN-17-e13140-s001.docx]

Supplementary material

## Maternal diet during pregnancy and its social determinants: the influence of migration status in the ELFE cohort.

## Supplementary Figure 1. Participant flow chart

## Supplementary Table 1, online. Benchmarks used for the Diet Quality score

| **Food group** | **Recommendation** | **Criterion used** |
| --- | --- | --- |
| Fruits and vegetables | ≥ 5 times/day | ≥ 5 times/day |
| Legumes | More than once a week | ≥ 2 times /week |
| Starchy foods | At each meal | 3 to 6 times /day |
| Whole-meal cereals | Increase the proportion of whole-grain cereals | Whole-grain bread/total bread ≥ 2/3 |
| Nuts | A small handful per day | 20 to 30 g/day |
| Dairy products | ≥ 3 times/day | ≥ 3 times/day |
| Meat/Fish/Egg | 1 to 2 times/day | 1 to 2 times/day |
| Fish | 2 times/week | 2 to 3 times/week |
| Red meat | < 500 g/week | < 500 g/week |
| Pork meat products | < 25 g/day | < 175 g/week |
| Added fat | Favor vegetable fats | Vegetable fat/Total fat ≥ 50% |
| Water | ≥ 1.3 L/day | ≥ 1300 mL/day |
| Sweetened beverages | Less than one drink per day | ≤ 250 mL/day |
| Coffee | ≤ 3 times/day | ≤ 3 times/day |
| Tea | ≤ 1 L/day | ≤ 1000 mL/day |
| Alcoholic beverages | Never | Never |
| Soy | ≤ 1 time/day | ≤ 1 time/day |

# Supplementary Methods - FFQ validation study

## Methods

### 24-hour recalls

A dietician contacted study participants by telephone to obtain a 24-hour recall each month during the last trimester of pregnancy (total of 3 attempted). Efforts were made to obtain one of the 24-hour recalls on a weekend, out of the three planned. The interviews were meal-sequence based and involved a detailed assessment and description of the foods and beverages consumed. Participants had to describe qualitatively and quantitatively all items consumed during the previous day, using household measures, to allow the dietician to estimate food and beverage intake. Three 24-hour recalls were available for 37 participants, two for 16 women, and only one for 3 (because they either could not be reached or gave birth preterm). The 24-hour recall data were sent to the Paris center to estimate food, energy, and macronutrient intake with the same food composition data as that utilized for the FFQ.

### FFQ validation analyses

Means and standard deviations for daily food and nutrient intakes were determined from both the FFQ and the 24-hour recalls. To take the unequal number of days for the 24-hour recall into account, within-person ($S_{w}^{2}$) and between-person ($S_{b}^{2}$) daily variances and their ratio were computed for each nutrient and food groups using a mixed model with a random coefficient for the subject and a compound symmetry variance-covariance matrix. Intraclass correlation coefficients were calculated with the formula S_b_^2^/(S_b_^2^+S_w_^2^). Pearson correlation coefficients were computed for intake of nutrients and Spearman correlation coefficients for food groups. Disattenuated correlation coefficients were also estimated to correct the correlation coefficient for the large variability of daily food intake ^54^, obtained from crude coefficients by multiplication by the factor: $\sqrt{1+\frac{R}{n}}$ where R is the ratio S_w_^2^/S_b_^2^ and n is the average number of 24-hour recalls per subject (2.61 in our study). Nutrient and food intake data were adjusted for energy with the residual method ^55^.

Study participants were classified into quintile categories of energy, macronutrient, and food intakes based on the distribution of data for both the FFQ and the average of the 24-hour recalls. The proportions of subjects correctly classified (if classified into same or adjacent quintiles) or grossly misclassified (if classified into opposing quintiles) was estimated.

## Results

For most nutrients examined (Supplementary table 3), the FFQ nutrient intake estimates were higher than those from the 24-hour recalls by 4% (for lipids) to 28% (for fibre). For monosaturated and polyunsaturated fatty acids, the FFQ estimates were lower by 14% and 5%, respectively. The low intraclass correlation coefficients, especially for fat intake, underline the large day-to-day variability of intakes. The crude Pearson correlation coefficient between estimates from FFQ and from 24-hour recalls ranged from 0.22 (fiber) to 0.54 (polyunsaturated fatty acids). The adjusted correlation coefficient for the two methods varied from 0.21 (monosaturated fatty acids) to 0.59 (fiber). The disattenuated correlation coefficient for the two methods improved for all nutrients (versus crude and energy-adjusted coefficients) and ranged from 0.37 (monosaturated fats) to 0.76 (fibre). The FFQ and repeated 24-hour recalls during the last trimester of pregnancy corresponded well, classifying most women into the same or adjacent quintiles. The proportion of women grossly misclassified (into opposite quintiles) was limited: for most nutrients examined, the rate was less than 6 %; for fiber intake, it was 7%. For most food groups examined, less than 5% of women were classified into opposite quintiles; however, for meat, as well as pasta/rice/potatoes groups, this rate was 7%.

## Supplementary Table 2. Consistency between nutrient or food group intake estimated by both the ELFE food frequency questionnaire and by 24-hour recalls (n=56)

|  |  | Daily intake  mean (sd) | |  |  | Daily variability  of 24-hR estimates | |  | Pearson's correlation between FFQ and 24-hR | | |  | Percentage of subjects classified into | | |
| --- | --- | --- | --- | --- | --- | --- | --- | --- | --- | --- | --- | --- | --- | --- | --- |
|  |  | FFQ | 24-hR | FFQ  (%24-hR)^†^ |  | S_w_^2^/S_b_^2 ‡^ | Intra class correlation coefficient |  | Crude | Adjusted  for energy | De-attenuated ^§^ |  | same  quintile | adjacent  quintiles | opposite  quintiles |
| Nutrients | |  |  |  |  |  |  |  |  |  |  |  |  |  |  |
|  | Energy (Kcal/d) | 2300 (714) | 2081 (496)* | 110 |  | 1.84 | 0.35 |  | 0.26 |  |  |  | 58.9 | 35.7 | 5.4 |
|  | Protein (g/d) | 97 (36) | 80 (20)* | 121 |  | 2.54 | 0.28 |  | 0.23 | 0.47 | 0.66 |  | 62.5 | 32.1 | 5.4 |
|  | Lipid (g/d) | 91 (34) | 88 (25) | 104 |  | 3.61 | 0.22 |  | 0.35 | 0.35 | 0.54 |  | 57.1 | 37.5 | 5.4 |
|  | Saturated fatty acid (g/ d) | 39 (15) | 37 (13) | 106 |  | 2.40 | 0.29 |  | 0.31 | 0.47 | 0.66 |  | 55.4 | 42.9 | 1.8 |
|  | Monosaturated fatty acid (g/ d) | 27 (12) | 32 (9)* | 86 |  | 5.54 | 0.15 |  | 0.32 | 0.21 | 0.37 |  | 62.5 | 33.9 | 3.6 |
|  | Polyunsaturated fatty acid (g/d) | 10 (4) | 11 (4) | 95 |  | 6.17 | 0.14 |  | 0.54 | 0.35 | 0.66 |  | 76.8 | 21.4 | 1.8 |
|  | Cholesterol (g/d) | 428 (213) | 341 (138)* | 126 |  | 4.29 | 0.19 |  | 0.22 | 0.26 | 0.43 |  | 57.1 | 42.9 | 0.0 |
|  | Carbohydrate (g/d) | 272 (80) | 242 (66)* | 112 |  | 1.36 | 0.42 |  | 0.25 | 0.34 | 0.42 |  | 57.1 | 37.5 | 5.4 |
|  | Sugar (g/d) | 140 (54) | 114 (37)* | 123 |  | 1.17 | 0.46 |  | 0.41 | 0.34 | 0.41 |  | 62.5 | 35.7 | 1.8 |
|  | Fiber (g/d) | 24 (7) | 19 (6)* | 128 |  | 1.75 | 0.36 |  | 0.22 | 0.59 | 0.76 |  | 60.7 | 32.1 | 7.1 |
| Food groups (g/day) | |  |  |  |  |  |  |  |  |  |  |  |  |  |  |
|  | Organ meats | 15 (27) | 14 (31)* | 109 |  | 9.00 | 0.10 |  | 0.23 | 0.17 | 0.35 |  | 60.7 | 39.3 | 0.0 |
|  | Eggs | 38 (36) | 11 (22)* | 342 |  | 21.21 | 0.05 |  | 0.19 | 0.23 | 0.68 |  | 58.9 | 39.3 | 1.8 |
|  | Meat | 102 (70) | 95 (73) | 107 |  | 3.63 | 0.22 |  | 0.10 | 0.23 | 0.35 |  | 55.4 | 37.5 | 7.1 |
|  | Fish and shellfish | 43 (60) | 34 (51)* | 129 |  | 7.83 | 0.11 |  | 0.55 | 0.49 | 0.97 |  | 76.8 | 23.2 | 0.0 |
|  | Cheese | 42 (41) | 33 (31) | 128 |  | 2.98 | 0.25 |  | 0.42 | 0.48 | 0.70 |  | 69.6 | 26.8 | 3.6 |
|  | Yoghurt | 143 (127) | 132 (110) | 108 |  | 1.48 | 0.40 |  | 0.45 | 0.42 | 0.52 |  | 75.0 | 23.2 | 1.8 |
|  | Dairy dessert | 46 (63) | 34 (47)* | 135 |  | 4.43 | 0.18 |  | 0.51 | 0.47 | 0.76 |  | 80.4 | 19.6 | 0.0 |
|  | Sugar and confectionery | 32 (24) | 30 (21) | 108 |  | 2.26 | 0.31 |  | 0.60 | 0.39 | 0.53 |  | 75.0 | 25.0 | 0.0 |
|  | Milk | 198 (193) | 151 (152) | 131 |  | 0.78 | 0.56 |  | 0.77 | 0.65 | 0.74 |  | 89.3 | 10.7 | 0.0 |
|  | Bread | 108 (58) | 96 (48) | 113 |  | 1.77 | 0.36 |  | 0.21 | 0.31 | 0.39 |  | 57.1 | 41.1 | 1.8 |
|  | Cereals | 9 (16) | 11 (20) | 83 |  | 1.03 | 0.49 |  | 0.56 | 0.38 | 0.45 |  | 73.2 | 26.8 | 0.0 |
|  | Pasta, rice, and potatoes | 145 (97) | 112 (77) | 130 |  | 4.95 | 0.17 |  | 0.25 | 0.28 | 0.47 |  | 60.7 | 32.1 | 7.1 |
|  | Cakes, pastries, biscuits | 57 (58) | 88 (98) | 65 |  | 2.37 | 0.30 |  | 0.31 | 0.12 | 0.16 |  | 71.4 | 23.2 | 5.4 |
|  | Fruits | 246 (209) | 215 (137) | 115 |  | 1.22 | 0.45 |  | 0.34 | 0.35 | 0.42 |  | 66.1 | 32.1 | 1.8 |
|  | Vegetables and legumes | 267 (153) | 223 (129) | 120 |  | 5.75 | 0.15 |  | 0.34 | 0.33 | 0.59 |  | 60.7 | 39.3 | 0.0 |
|  | Sweet beverages | 262 (263) | 148 (162)* | 177 |  | 0.68 | 0.59 |  | 0.54 | 0.46 | 0.52 |  | 69.6 | 26.8 | 3.6 |
|  | Ready-prepared dishes | 55 (60) | 97 (99) | 56 |  | 10.17 | 0.09 |  | 0.34 | 0.29 | 0.63 |  | 58.9 | 37.5 | 3.6 |

FFQ: Food frequency questionnaire; 24-hR: 24-hour recall

* Significantly different from the value of the 24-HR: Student’s test for nutrients and Wilcoxon’s test for food groups, P<0.05.

^†^: ratio of nutrient intake by FFQ and 24-hour recall methods; represents the degree of concordance between FFQ compared to 24-hour recall. A ratio of 100 represents identical mean values obtained by the two methods.

^‡^: ratio of within-person ($S_{w}^{2}$) and between-person ($S_{b}^{2}$) variance for the 24-hour recalls

^§^: Coefficient corrected for within-person variation in 24-hour recalls

## Supplementary Table 3. Bivariate associations between familial characteristics and maternal diet during pregnancy (n=12,048): the ELFE study, 2011

|  |  | Diet quality score | PANDiet score | PCA pattern 1:  Western | PCA pattern 2:  Healthy | PCA pattern 3:  Traditional slice of bread | PCA pattern 4:  Processed products | PCA pattern 5:  Milk & breakfast |
| --- | --- | --- | --- | --- | --- | --- | --- | --- |
| Maternal age at delivery | |  |  |  |  |  |  |  |
|  | < 25 y | -0.52 [-0.60 ; -0.44]^†^ | -1.14 [-1.79 ; -0.50] | 0.36 [0.29 ; 0.43] | -0.45 [-0.52 ; -0.39] | -0.26 [-0.33 ; -0.20] | -0.16 [-0.23 ; -0.09] | 0.02 [-0.05 ; 0.09] |
|  | 25-29 y | 0 [Ref] | 0 [Ref] | 0 [Ref] | 0 [Ref] | 0 [Ref] | 0 [Ref] | 0 [Ref] |
|  | 30-34 y | 0.25 [0.20 ; 0.30] | 0.57 [0.18 ; 0.96] | -0.09 [-0.13 ; -0.05] | 0.33 [0.29 ; 0.37] | 0.20 [0.16 ; 0.24] | 0.15 [0.11 ; 0.20] | -0.12 [-0.17 ; -0.08] |
|  | 35 y or more | 0.45 [0.39 ; 0.51] | 1.43 [0.98 ; 1.87] | -0.18 [-0.22 ; -0.13] | 0.61 [0.57 ; 0.66] | 0.21 [0.16 ; 0.25] | 0.19 [0.14 ; 0.24] | -0.24 [-0.29 ; -0.19] |
| Maternal education level | |  |  |  |  |  |  |  |
|  | Up to lower secondary | -0.77 [-0.90 ; -0.64] | -1.78 [-2.77 ; -0.78] | 0.62 [0.51 ; 0.72] | -0.74 [-0.85 ; -0.64] | -0.64 [-0.75 ; -0.54] | -0.31 [-0.42 ; -0.21] | -0.15 [-0.26 ; -0.05] |
|  | Upper secondary | -0.61 [-0.67 ; -0.55] | -2.30 [-2.75 ; -1.85] | 0.45 [0.41 ; 0.50] | -0.60 [-0.64 ; -0.55] | -0.39 [-0.44 ; -0.34] | -0.26 [-0.31 ; -0.21] | -0.16 [-0.21 ; -0.11] |
|  | Intermediate | -0.30 [-0.37 ; -0.24] | -1.83 [-2.31 ; -1.35] | 0.19 [0.14 ; 0.24] | -0.35 [-0.40 ; -0.30] | -0.17 [-0.22 ; -0.12] | -0.07 [-0.12 ; -0.02] | -0.05 [-0.11 ; 0.00] |
|  | 3-y university degree | -0.09 [-0.16 ; -0.02] | -0.55 [-1.06 ; -0.04] | 0.12 [0.07 ; 0.17] | -0.15 [-0.20 ; -0.10] | -0.04 [-0.09 ; 0.02] | -0.06 [-0.11 ; 0.00] | 0.02 [-0.04 ; 0.07] |
|  | At least 5-y university degree | 0 [Ref] | 0 [Ref] | 0 [Ref] | 0 [Ref] | 0 [Ref] | 0 [Ref] | 0 [Ref] |
| Maternal migration status | |  |  |  |  |  |  |  |
|  | Immigrant | 0.35 [0.27 ; 0.44] | 4.20 [3.57 ; 4.83] | -0.01 [-0.08 ; 0.05] | 0.36 [0.29 ; 0.42] | -0.65 [-0.71 ; -0.58] | -0.31 [-0.38 ; -0.25] | 0.24 [0.17 ; 0.30] |
|  | Descendant of at least one immigrant | 0.10 [0.03 ; 0.17] | 1.60 [1.05 ; 2.14] | 0.04 [-0.02 ; 0.10] | 0.04 [-0.02 ; 0.10] | -0.32 [-0.38 ; -0.27] | -0.23 [-0.29 ; -0.18] | 0.03 [-0.03 ; 0.09] |
|  | Majority population | 0 [Ref] | 0 [Ref] | 0 [Ref] | 0 [Ref] | 0 [Ref] | 0 [Ref] | 0 [Ref] |
| Single parenthood | |  |  |  |  |  |  |  |
|  | No | 0 [Ref] | 0 [Ref] | 0 [Ref] | 0 [Ref] | 0 [Ref] | 0 [Ref] | 0 [Ref] |
|  | Yes | -0.43 [-0.55 ; -0.30] | -0.47 [-1.42 ; 0.47] | 0.33 [0.23 ; 0.43] | -0.26 [-0.36 ; -0.16] | -0.40 [-0.50 ; -0.30] | 0 [-0.11 ; 0.10] | -0.03 [-0.13 ; 0.07] |
| Older children in household | |  |  |  |  |  |  |  |
|  | ELFE child is the first child | 0 [Ref] | 0 [Ref] | 0 [Ref] | 0 [Ref] | 0 [Ref] | 0 [Ref] | 0 [Ref] |
|  | At least one older child | 0.08 [0.03 ; 0.12] | -0.06 [-0.39 ; 0.27] | 0.15 [0.12 ; 0.19] | 0.11 [0.07 ; 0.14] | 0.10 [0.06 ; 0.13] | 0.05 [0.01 ; 0.08] | -0.26 [-0.29 ; -0.22] |
| Maternal job status during pregnancy | |  |  |  |  |  |  |  |
|  | Employed | 0 [Ref] | 0 [Ref] | 0 [Ref] | 0 [Ref] | 0 [Ref] | 0 [Ref] | 0 [Ref] |
|  | Unemployed | -0.20 [-0.27 ; -0.13] | -0.54 [-1.06 ; -0.02] | 0.26 [0.20 ; 0.31] | -0.22 [-0.28 ; -0.17] | -0.19 [-0.24 ; -0.13] | -0.23 [-0.28 ; -0.17] | 0.06 [0.00 ; 0.12] |
|  | Not in the labour force | -0.18 [-0.24 ; -0.11] | 0.60 [0.10 ; 1.09] | 0.27 [0.22 ; 0.33] | -0.13 [-0.18 ; -0.07] | -0.34 [-0.39 ; -0.28] | -0.22 [-0.27 ; -0.17] | -0.02 [-0.08 ; 0.03] |
| Household income | |  |  |  |  |  |  |  |
|  | <=€750/mo/cu^‡^ | -0.18 [-0.27 ; -0.09] | 1.03 [0.33 ; 1.72] | 0.34 [0.27 ; 0.42] | -0.11 [-0.18 ; -0.04] | -0.46 [-0.53 ; -0.39] | -0.22 [-0.30 ; -0.15] | 0 [-0.08 ; 0.07] |
|  | 751 - €1111/mo/cu^§^ | -0.11 [-0.19 ; -0.04] | 0.18 [-0.38 ; 0.73] | 0.19 [0.13 ; 0.24] | -0.10 [-0.16 ; -0.04] | -0.22 [-0.27 ; -0.16] | -0.17 [-0.23 ; -0.11] | -0.07 [-0.13 ; -0.01] |
|  | 1112 - €1500/mo/cu^¶^ | 0 [Ref] | 0 [Ref] | 0 [Ref] | 0 [Ref] | 0 [Ref] | 0 [Ref] | 0 [Ref] |
|  | 1501 - €1944/mo/cu^††^ | 0.13 [0.08 ; 0.19] | 0.52 [0.08 ; 0.96] | -0.14 [-0.18 ; -0.09] | 0.11 [0.06 ; 0.16] | 0.13 [0.08 ; 0.17] | 0.06 [0.01 ; 0.10] | 0.04 [-0.01 ; 0.08] |
|  | 1945 - €2500/mo/cu^‡‡^ | 0.28 [0.21 ; 0.35] | 0.87 [0.37 ; 1.37] | -0.26 [-0.31 ; -0.21] | 0.27 [0.22 ; 0.33] | 0.20 [0.15 ; 0.26] | 0.14 [0.09 ; 0.20] | 0.13 [0.08 ; 0.18] |
|  | > €2500 /mo/cu^§§^ | 0.37 [0.30 ; 0.45] | 2.15 [1.56 ; 2.75] | -0.36 [-0.42 ; -0.29] | 0.43 [0.36 ; 0.49] | 0.20 [0.14 ; 0.27] | 0.18 [0.12 ; 0.25] | 0.05 [-0.01 ; 0.12] |
| Area of residence | |  |  |  |  |  |  |  |
|  | Paris region | 0 [Ref] | 0 [Ref] | 0 [Ref] | 0 [Ref] | 0 [Ref] | 0 [Ref] | 0 [Ref] |
|  | North | -0.31 [-0.40 ; -0.22] | -1.49 [-2.16 ; -0.82] | 0.36 [0.29 ; 0.43] | -0.49 [-0.56 ; -0.42] | -0.11 [-0.18 ; -0.04] | -0.19 [-0.26 ; -0.12] | -0.18 [-0.25 ; -0.10] |
|  | East | -0.18 [-0.26 ; -0.09] | -1.26 [-1.91 ; -0.62] | 0.14 [0.07 ; 0.20] | -0.18 [-0.25 ; -0.11] | 0 [-0.06 ; 0.07] | -0.16 [-0.23 ; -0.09] | -0.21 [-0.28 ; -0.14] |
|  | Paris Basin - East | -0.19 [-0.28 ; -0.10] | -1.26 [-1.95 ; -0.58] | 0.28 [0.21 ; 0.35] | -0.24 [-0.31 ; -0.17] | 0.10 [0.03 ; 0.17] | -0.12 [-0.20 ; -0.05] | -0.13 [-0.20 ; -0.05] |
|  | Paris Basin - West | -0.07 [-0.16 ; 0.02] | -0.91 [-1.59 ; -0.24] | 0.19 [0.12 ; 0.26] | -0.11 [-0.18 ; -0.04] | 0.18 [0.11 ; 0.25] | -0.08 [-0.15 ; -0.01] | -0.04 [-0.12 ; 0.03] |
|  | West | 0.03 [-0.05 ; 0.11] | -0.85 [-1.43 ; -0.27] | 0.15 [0.09 ; 0.21] | -0.02 [-0.09 ; 0.04] | 0.29 [0.23 ; 0.35] | 0.04 [-0.03 ; 0.10] | -0.09 [-0.15 ; -0.03] |
|  | Southwest | 0.03 [-0.07 ; 0.12] | 0.05 [-0.64 ; 0.74] | 0.06 [-0.01 ; 0.13] | 0.07 [0.00 ; 0.14] | 0.12 [0.04 ; 0.19] | -0.06 [-0.14 ; 0.01] | -0.11 [-0.19 ; -0.04] |
|  | Southeast | 0.04 [-0.05 ; 0.12] | -0.73 [-1.35 ; -0.11] | -0.02 [-0.09 ; 0.04] | 0.00 [-0.06 ; 0.07] | 0.17 [0.11 ; 0.24] | -0.13 [-0.20 ; -0.07] | -0.10 [-0.17 ; -0.03] |
|  | Mediterranean | 0.04 [-0.05 ; 0.12] | 0.33 [-0.30 ; 0.95] | 0.11 [0.04 ; 0.17] | -0.02 [-0.09 ; 0.05] | -0.01 [-0.07 ; 0.06] | -0.12 [-0.19 ; -0.05] | -0.09 [-0.15 ; -0.02] |
| Enrollment wave | |  |  |  |  |  |  |  |
|  | Wave 1 | 0.02 [-0.05 ; 0.09] | 0.34 [-0.17 ; 0.85] | -0.02 [-0.07 ; 0.03] | 0.04 [-0.02 ; 0.09] | 0.05 [0.00 ; 0.10] | 0.06 [0.00 ; 0.11] | 0.08 [0.03 ; 0.14] |
|  | Wave 2 | 0.03 [-0.03 ; 0.09] | -0.01 [-0.45 ; 0.43] | 0.02 [-0.03 ; 0.06] | 0.01 [-0.03 ; 0.06] | -0.03 [-0.07 ; 0.02] | 0.10 [0.05 ; 0.14] | -0.10 [-0.15 ; -0.05] |
|  | Wave 3 | -0.06 [-0.12 ; -0.01] | -0.51 [-0.93 ; -0.08] | 0.03 [-0.02 ; 0.07] | -0.03 [-0.07 ; 0.02] | -0.03 [-0.08 ; 0.01] | 0.09 [0.04 ; 0.13] | -0.11 [-0.16 ; -0.07] |
|  | Wave 4 | 0 [Ref] | 0 [Ref] | 0 [Ref] | 0 [Ref] | 0 [Ref] | 0 [Ref] | 0 [Ref] |
| Maternity size | |  |  |  |  |  |  |  |
|  | 145-699 deliveries/y | -0.09 [-0.23 ; 0.04] | -0.86 [-1.88 ; 0.16] | 0.04 [-0.06 ; 0.15] | -0.09 [-0.20 ; 0.02] | 0.10 [0.00 ; 0.21] | -0.06 [-0.17 ; 0.05] | -0.10 [-0.21 ; 0.01] |
|  | 700-1,009 deliveries/y | -0.11 [-0.19 ; -0.02] | 0.17 [-0.45 ; 0.80] | 0.18 [0.12 ; 0.25] | -0.05 [-0.12 ; 0.02] | 0.04 [-0.02 ; 0.11] | -0.04 [-0.10 ; 0.03] | -0.08 [-0.15 ; -0.01] |
|  | 1,010-1,418 deliveries/y | -0.17 [-0.24 ; -0.11] | -0.70 [-1.21 ; -0.20] | 0.08 [0.03 ; 0.13] | -0.12 [-0.17 ; -0.06] | -0.02 [-0.07 ; 0.04] | -0.04 [-0.09 ; 0.02] | -0.05 [-0.11 ; 0.00] |
|  | 1,422-2,187 deliveries/y | -0.06 [-0.11 ; -0.01] | -0.15 [-0.54 ; 0.23] | 0.05 [0.01 ; 0.09] | -0.04 [-0.08 ; 0.01] | 0.02 [-0.02 ; 0.06] | -0.03 [-0.07 ; 0.01] | -0.03 [-0.07 ; 0.01] |
|  | 2,197-5,215 deliveries/y | 0 [Ref] | 0 [Ref] | 0 [Ref] | 0 [Ref] | 0 [Ref] | 0 [Ref] | 0 [Ref] |

cu: consumption unit

^†^ Values are estimates [95% CI] from simple linear regressions ^‡^ corresponding to ≤ $ 815 /month; ^§^ corresponding to $ 816 – 1,207 /month; ^¶^ corresponding to $ 1,208 – 1,630 /month; ^††^ corresponding to $ 1,631 – 2,113 /month; ^‡‡^ corresponding to $ 2,114 – 2,717 /month; ^§§^ corresponding to > $ 2,717 /month.
